# Postpartum Depression: Plaguing the Joy of New Mothers

**DOI:** 10.1192/j.eurpsy.2024.1685

**Published:** 2024-08-27

**Authors:** G. Sonkar, M. Sonkar

**Affiliations:** ^1^Anaesthesia, Ram Manohar Lohia Hospital; ^2^Mental Health First Aider, Noida, India

## Abstract

**Introduction:**

Depression is a significant global mental health problem and is very common compared to how it is perceived. In 2020 alone, 264 million people globally suffered from depression and its different forms as per the World Health Organisation. It is a leading cause of disability in individuals, affecting their ability to perform their daily chores, work, study, and even maintain relationships. The impact of depression is deeper and affects families, the economy, health care systems, and so on.

In India, the problem is grave and leads to serious consequences, thanks to the stigma and unawareness attached to mental health disorders. It is estimated that India has one of the highest rates of depression in the world, but it is hardly acknowledged.

Post-Partum Depression (PPD) is the most neglected and unreported subtype of depression in India. Globally, 1 in every 7 women suffers from Post Partum Depression. India is such a diverse country in terms of prevalence varies from 15% to 25% based on region, population, cultural and social expectations, economic status, living standard, climate factors, and others.

**Objectives:**

The objective of the study is to spread awareness, identify the risk factors, root cause analysis of risk factors, possible solutions, and treatments.

**Methods:**

This study is conducted to capture the awareness level of PPD in females across different ages, regions, income classes, cultures, working statuses, and societies. This is carried out using a detailed yet anonymous survey, it captures the demography, knowledge of signs and symptoms of PPD, personal experiences, attitudes, expected support for PPD, and awareness of possible healthcare options. The result of the study tries to understand and conclude the most common risk factors, groups at highest risk, a root cause analysis of the risk factors, and possible solutions and treatments.

**Results:**

PPD occurs in the postnatal period, typically within the first year after childbirth. This condition can have a significant impact on the new mother and the infant’s well-being. The mother’s ability to take care of the child and herself is hugely impacted, impacting the child’s development and family dynamics negatively. Pushing to the limits, certain communities that believe in superstition and taboo often take PPD as an excuse to blame the mother resulting in the extremities like suicides.

**Conclusions:**

PPD occurs in the postnatal period, typically within the first year after childbirth. This condition can have a significant impact on the new mother and the infant’s well-being. The mother’s ability to take care of the child and herself is hugely impacted, impacting the child’s development and family dynamics negatively. Pushing to the limits, certain communities that believe in superstition and taboo often take PPD as an excuse to blame the mother resulting in the extremities like suicides.

**Disclosure of Interest:**

None Declared

